# Polymorphism +17 C/G in Matrix Metalloprotease MMP8 decreases lung cancer risk

**DOI:** 10.1186/1471-2407-8-378

**Published:** 2008-12-19

**Authors:** Patricia González-Arriaga, M Felicitas López-Cima, Ana Fernández-Somoano, Teresa Pascual, Manuel G Marrón, Xose S Puente, Adonina Tardón

**Affiliations:** 1 Departamento de Medicina, Unidad de Epidemiología Molecular del Instituto Universitario de Oncología, Universidad de Oviedo, 33006 Oviedo, Spain and CIBER Epidemiología y Salud Pública (CIBERESP), Spain; 2 Servicio de Neumología, Hospital de Cabueñes, Gijón, Spain; 3 Departamento de Bioquímica y Biología Molecular, Instituto Universitario de Oncología del Principado de Asturias, Universidad de Oviedo, 33006 Oviedo, Spain

## Abstract

**Background:**

Matrix metalloproteases (MMPs) constitute a family of enzymes capable of degrading different components of the extracellular matrix and are implicated in the invasion of tumor cells through the basement membrane. Polymorphisms in MMP genes may result in changes in the expression of MMPs being associated with the development and progression of cancer. We have investigated the association between three polymorphisms (-1607 1G/2G, +17 C/G and -77 A/G) in the human collagenases MMP1, MMP8 and MMP13 and the risk of development or progression of lung cancer.

**Methods:**

A hospital-based case-control study was designed including 501 lung cancer patients and 510 controls matched. Genotypes were determined by PCR-RFLP. Results were analyzed using unconditional logistic regression, Cox's proportional hazard regression, and the Kaplan-Meier method.

**Results:**

The MMP1 and MMP13 promoter polymorphisms were not associated with lung cancer risk, while the C/G polymorphism in MMP8 was associated with a statistically significant decreased risk of developing lung cancer (ORadj = 0.65; 95%CI = 0.45–0.93). The Kaplan-Meier analysis showed that the polymorphisms in MMP1, MMP8 and MMP13 not seem to modify the overall survival. Multivariate analysis revealed that MMP1, MMP8 and MMP13 polymorphisms are not independent prognostic factors for overall survival.

**Conclusion:**

This study suggests that the polymorphism in MMP8 is associated with a decreased lung cancer risk, which can be used as a prognostic marker in lung cancer.

## Background

Lung cancer constitutes one of the leading causes of death in industrialized countries, and its incidence is rapidly growing in developing nations worldwide. Although tobacco smoke and other environmental pollutants are responsible for more than 80–90% of the cases in men [1], it is well established that less than 10–15% of smokers develop lung cancer, indicating that other factors might contribute to the development of this disease [2,3]. In this regard, the availability of the human genome sequence has revealed the existence of numerous polymorphisms that affect both coding and non-coding regions [4], which might contribute to differences in the individual susceptibility to develop cancer [5-7].

One of the main characteristics of cancer cells is their ability to proliferate, invade the surrounding tissues and migrate to distant organs and form metastasis, thereby resulting in the emergence of disseminated metastases, which remains the primary cause of mortality in cancer patients [8-10]. Matrix metalloproteases (MMPs) constitute a group with the ability to cleave most components of the extracellular matrix, including collagen, laminin, fibronectin, proteoglycans or elastin, among others [11,12]. The expression of these MMPs by tumor cells might contribute to increasing the invasive potential of tumoral cells by allowing the remodeling of the extracellular matrix. In this sense, overexpression of collagenase-1 (MMP1) and collagenase-3 (MMP13) has been associated with more aggressive tumors and poor prognosis in different tumor types [13,14]. Furthermore, MMP1 expression has been found to be an important marker of metastasis in breast cancer cells, confirming the importance of MMPs in tumor growth and invasion [13,15].

Polymorphisms in the regulatory regions of MMPs have been associated with changes in the expression level of these genes in different human diseases [16-18]. In fact, the -1607 1G/2G polymorphism in the promoter region of MMP1 creates an Ets binding site which increases the promoter activity of this gene [19]. Thus, the 2G allele of MMP1 has significantly higher transcriptional activity than the 1G allele and has been associated with an increased risk of common cancers, including oral, colorectal, renal and head and neck [17,20-22]. Furthermore, in colorectal and ovarian cancer, the presence of the 2G allele in the MMP1 gene was significantly associated with poorer survival of patients with cancer [23,24]. On the other hand, the -77 A/G polymorphism in the promoter region of MMP13, which modifies a PEA3 binding site resulting in reduced transcriptional activity of this gene [25], might contribute to reduce the risk of developing cancer. However, most of the epidemiological studies do not support this biological evidence. Two recent studies have shown no association between the -77 A/G polymorphism and the risk of developing breast and nasopharyngeal cancer [26-28]. Finally, although recent studies in animal models have shown that mutant mice deficient in MMP8 are more susceptible to develop skin cancer, suggesting that MMP8 has a protective function against tumor developments [29,30], there are no epidemiological studies to analyze the association between polymorphisms in the promoter region of MMP8 and the susceptibility to develop cancer.

In this study, we reasoned that polymorphisms in the regulatory regions of the three human collagenases (MMP1, MMP8 and MMP13), affecting the expression level of these genes, might influence the risk and survival of lung cancer patients.

## Methods

### Study subjects

The detailed methods of recruiting participants for this hospital-based case-control study have been described elsewhere [31-33]. Briefly, a total of 501 incident cases of histologically confirmed lung cancer were recruited in two main hospitals of Asturias in Northern Spain [Cabueñes Hospital in Gijón (54%) and San Agustín Hospital in Avilés (46%)], following an identical protocol from October 2000 to April 2006. 510 controls were selected from patients admitted to participating hospitals for diagnoses believed to be unrelated to the exposures of interest, individually matched to the cases in ethnicity, gender and age (± 5 years). The main specific pathologies of the final controls selected were: 41.1% inguinal and abdominal hernias (ICD-9: 550–553), 32.5% injuries (ICD-9: 800–848, 860–869, 880–897), 8.8% appendicitis (ICD-9: 540), and 13.3% intestinal obstructions (ICD-9: 560, 569, 574). The study was approved by the ethical committee of the hospitals, and written consent was obtained from each participant.

### Data collection

Information on known or potential risk factors for lung cancer was collected personally through computer-assisted questionnaires by trained interviewers during the first hospital admission for diagnosis. Structured questionnaires collected information on age, gender, sociodemographic characteristics, recent and prior tobacco use, and personal and family history of cancer (first-degree relatives) from each participant. A total of 93.8% eligible cases and 98.5% of eligible controls agreed to participate in the study and were interviewed. Reasons for eligibility are: pathology confirmation, residence, reassignments, and mental handicap. Of the 759 cases and 593 controls interviewed, 741 (97.6%) cases and 556 (93.8%) controls provided a blood or buccal cell sample for DNA extraction. Fifty-five individuals were excluded, seventeen individuals (5 cases and 12 controls) because of low amounts of DNA and thirty-eight (15 cases and 23 controls) by difficulties in the genotyping. Thirty seven individuals (26 cases and 11 controls) with missing information in the questionnaires and 194 cases without matched controls were also excluded from these analyses. Thus, the final study population available for this study was 501 cases and 510 controls, all of whom were Caucasian.

### Genotype determination

The polymorphisms in the promoters of MMP genes analyzed in this study are shown in Table 1. The polymerase chain reaction (PCR) combined with restriction fragment length polymorphism (RFLP) was used to determine the MMPs genotypes. Genomic DNA used for the assay was extracted from peripheral blood samples (96.5% of total) or exfoliated buccal cells (3.5% of total) as previously described [34]. For quality control, genotyping was repeated randomly in at least 5% of the samples, and two of the authors independently reviewed all results. A quality control of 50 blood and mouthwash samples from the same participants ensured the reliability of genotyping results of mouthwash samples. In both quality controls no differences were found. PCR reactions were carried out in a total volume 10 μl containing 20 ng of genomic DNA, 0.25 mM each dNTPs (Ecogen, Biologia Molecular S.L.), 0.2 units Taq polymerase (Biotools, Inc.) and 2.5 pmol of each primer in 1 × PCR buffer (Sigma-Aldrich Co.). The details of primers and PCR conditions used for the amplification of MMPs are shown in Table 1. For MMP1, the reverse primer was designed to introduce a recognition site for the restriction enzyme *Alu*I (New England Biolabs, Inc.) by replacing a T with a G. A 5 μl aliquot of PCR products was digested overnight at 37°C with 0.4 units of the indicated restriction enzyme. After overnight digestion, the products were separated on agarose gels and stained with ethidium bromide (restriction enzyme are shown in Table 1). To verify that the data obtained by RFLP was coincident with the allele sequence, representative fragments were sequenced (data not shown).

**Table 1:** Details of PCR conditions and RFLPs studied

**Gene**	**Polymorphism**	**Primer sequence**	**PCR Conditions**	**Enzyme**
MMP1	-16071G/2G	(F) TGA CTT TTA AAA CAT AGT CTA TGT TCA (R) TCT TGG ATT GAT TTG AGA TAA GTC ATA gC	35 cycles: 94°C 30 s, 54°C 30 s, 72°C 30 s	*Alu*I
MMP8	+ 17C/G	(F) CTG TTG AAG GCC TAG AGC TGC TGC TCC (R) CAT CTT CTC TTC AAA CTC TAC CC	30 cycles: 94°C 45 s, 64°C 45 s, 72°C 1 min	*Dde*I
MMP13	-77A/G	(F) GAT ACG TTC TTA CAG AAG GGC (R) GAC AAA TCA TCT TCA TCA CC	30 cycles: 94°C 30 s, 54°C 30 s, 72°C 30 s	*Brs*I

### Statistical Analysis

Tests for Hardy-Weinberg equilibrium among controls were conducted using observed genotype frequencies and a χ^2 ^test with one degree of freedom. Univariate analysis was first performed to compare the distribution of age and gender and the frequencies of alleles and genotypes. The differences in the distribution between cases and controls were tested using the χ^2^, Fisher exact, and Mann-Whitney U-test, where appropriate. The crude odd ratios (ORs) were calculated by Wolf's method [35]. Multivariate unconditional logistic regression analysis with adjustment for age, gender, family history of cancer (first-degree relatives), and pack-years was performed to calculate adjusted ORs and 95% confidence intervals (CIs). The survival curves were constructed using the Kaplan-Meier method, and differences between the groups were tested by the log-rank method. The multivariate analysis of probable prognostic factors for survival was performed using Cox's proportional hazard regression analysis and relative risk with 95% confidence intervals, with adjustment for variables with statistical significance in univariate analysis. All statistical analyses were performed with STATA version 8 software. The sample size of our study for an allele frequency of 13% is enough to detect ORs greater than 0.65 with 84.5% power assuming a log-additive model, while for an allele frequency of 28% the power to detect an OR of 1.24 is 60%. However, the power to detect an OR greater than 1.00 for 51% allele frequencies is only 5%.

## Results

### Population Characteristics

The analysis included 501 lung cancer cases and 510 controls from the Caucasian population of Asturias, Northern Spain. The distributions of age, gender, smoking status, family history of cancer, histological types and clinical stages data for the cases among the study subjects are summarized in Table 2. There were no statistically significant differences among cases and controls in terms of mean age and gender distributions, suggesting that the frequency matching was adequate. Furthermore, the control population was consistent with Hardy-Weinberg equilibrium for all polymorphisms studied.

**Table 2:** Characteristics of lung cancer cases and control patients in a Spanish population

**Variable**	**Cases (n = 501)** **n (%)**	**Controls (n = 510)** **n (%)**	**P^a^**
Gender			
Male	441 (88.0)	440 (86.3)	
Female	60 (12.0)	70 (13.7)	0.406
			
Age (yrs), mean (SD)	64.7 (11.0)	63.6 (11.2)	0.137
			
Smoking Status			
Never	35 (7.0)	136 (26.7)	
Ever	466 (93.0)	374 (73.3)	< 0.001
Former	211 (45.9)	215 (59.9)	
Current	249 (54.1)	144 (40.1)	< 0.001
			
Pack-years ^b^, mean (SD)	63.0 (36.5)	39.4 (33.3)	< 0.001
			
Family history of cancer			
No	262 (54.5)	304 (60.7)	
Yes	219 (45.5)	197 (39.3)	0.049
Lung cancer	57 (12.3)	35 (7.2)	
Other cancers	144 (31.1)	147 (30.5)	0.020
			
Histological types			
Squamous cell carcinoma	202 (40.3)		
Adenocarcinoma	143 (28.5)		
Small cell carcinoma	81 (16.2)		
Non-differentiated	37 (7.4)		
Large cell carcinoma	15 (3.0)		
Others	8 (1.6)		
Clinical diagnosis	2 (0.4)		
Missing	13 (2.6)		
			
Clinical stages			
I	126 (25.1)		
II	28 (5.6)		
III	119 (23.8)		
IV	138 (27.5)		
LS (Limited stage)	39 (7.8)		
EE (Extend stage)	32 (6.4)		
Missing	19 (3.8)		

^a ^Two-sided χ^2 ^test and Mann-Whitney where appropriate

^b ^Pack-years for ever smokers

We have determined the frequency of 3 polymorphisms in 3 human collagenases implicated in the degradation of the extracellular matrix components and basement membranes in lung cancer patients and matching controls in order to evaluate their association with the risk and the survival of lung cancer.

### Analysis of the -1607 1G/2G polymorphism in the MMP1 gene

The distribution of MMP1 genotypes was very similar between cases and controls, with frequencies of 1G/1G, 1G/2G and 2G/2G genotypes of 25.5%, 49.5% and 25.0% in cases and 23.3%, 50.8% and 25.9% in controls, respectively. When we evaluated the association between MMP1 genotypes and lung cancer risk (Table 3), we found that individual with 2G/2G genotype and individual with 1G/2G or 2G/2G genotype (2G carriers) presented a lack of association with lung cancer risk (adjusted OR = 1.04; 95% CI = 0.68–1.58 and adjusted OR = 0.99; 95% CI = 0.71–1.40, respectively). When we carried out stratified analysis by selected variables [see Additional file 1], no association was found between the MMP1 polymorphism and lung cancer risk for age, gender, smoking status, and family history of cancer. However, stratified analysis by histological types, showed that the MMP1 2G/2G genotype potentially tends to increase the risk of developing small cell lung cancer (adjusted OR = 2.06; 95% CI = 0.94–4.51; *P *= 0.072) [see Additional file 2].

**Table 3:** Analysis of polymorphisms and lung cancer risk estimates

**MMPs**	**Genotype**	**Cases** **n(%)**	**Controls** **n(%)**	**Adjusted^a ^OR** **[95% CI]**	**P**	**P trend**
MMP1	1G/1G	128 (25.5)	119 (23.3)	Reference		
	1G/2G	248 (49.5)	259 (50.8)	0.97 [0.68–1.40]	0.884	
	2G/2G	125 (25.0)	132 (25.9)	1.04 [0.68–1.58]	0.859	0.857
	1G/2G + 2G/2G	373 (74.5)	391 (76.7)	0.99 [0.71–1.40]	0.975	0.975
						
MMP8	C/C	392 (79.7)	358 (75.2)	Reference		
	C/G + G/G	100 (20.3)	118 (24.8)	0.65 [0.45–0.93]	0.019	0.019
						
MMP13	A/A	248 (49.5)	267 (52.4)	Reference		
	A/G	208 (41.5)	197 (39.4)	1.24 [0.91–1.69]	0.174	
	G/G	45 (9.0)	42 (8.2)	1.23 [0.72–2.11]	0.452	0.200
	A/G + G/G	253 (50.5)	239 (47.6)	1.24 [0.92–1.66]	0.155	0.155

^a^Adjusted by age, gender, tobacco consumption (in pack-years: never, ≤ 31.5 and > 31.5), and family history of cancer

### Analysis of the -77 A/G polymorphism in the MMP13 gene

The frequency of the MMP13 -77G allele for cases and controls was 0.297 and 0.279 respectively, similar to the variant allele for MMP1. Similar to the 1G/2G polymorphism in the MMP1 promoter, the MMP13 G/G genotype presented a lack of association with lung cancer risk (adjusted OR = 1.23; 95% CI = 0.72–2.11) (Table 3). However, the stratified analysis [see Additional file 3] showed that the genotype A/G was significantly associated with an increased risk of developing lung cancer in former smokers (adjusted OR = 1.60; 95% CI = 1.05–2.45; *P *= 0.030). Looking at the histological types [see Additional file 2], the results showed a higher risk of developing small cell lung cancer for heterozygous and homozygous genotypes (adjusted OR = 1.94; 95% CI = 1.09–3.45; *P *= 0.024 and adjusted OR = 2.38; 95% CI = 1.01–5.65;*P *= 0.048, respectively).

### Analysis of the +17 C/G polymorphism in the MMP8 gene

In the case of the +17 C/G polymorphism in MMP8, we found that the frequency of the +17G allele was 0.130 in our population control. Due to the low number of individuals homozygous for the G/G genotype (8 cases and 6 controls), we examined the association between the presence of the G allele and lung cancer risk (Table 3). Thus, variant allele carriers showed a statistically significant decreased risk for developing lung cancer (adjusted OR = 0.65; 95% CI = 0.45–0.93; *P *= 0.019). In the stratified analysis we found a statistically significant decreased risk of lung cancer in male (adjusted OR = 0.63; 95% CI = 0.43–0.93; *P *= 0.021), ever smokers (adjusted OR = 0.69; 95% CI = 0.48–0.97; *P *= 0.034), and individual with family history of lung cancer (adjusted OR = 0.14; 95% CI = 0.03–0.64; *P *= 0.011) [see Additional file 4]. Looking at histological types [see Additional file 2], we observed that the G variant allele was significantly associated with a decreased risk for small cell carcinoma (adjusted OR = 0.43; 95% CI = 0.20–0.89; *P *= 0.023) and squamous cell carcinoma (adjusted OR = 0.51; 95% CI = 0.31–0.84; *P *= 0.008).

### Survival Analysis

We have furthermore evaluated the progression and survival of lung cancer patients. Survival questionnaires were collected for lung cancer patients who had been diagnosed at least 24 months earlier. Thus, a total of 376 eligible cases were selected until April of 2005. The overall survival rate was 22%. We used the Kaplan-Meier survival analysis to examine the relationship between polymorphisms in the promoter of the MMP genes and survival time.

**Figure 1:**
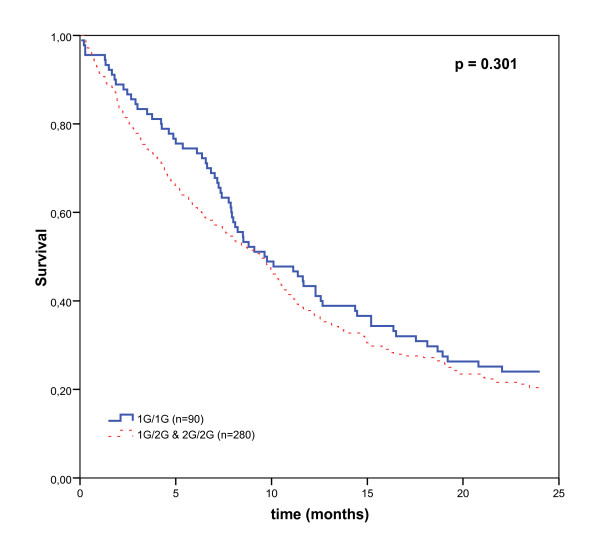
**Kaplan-Meier overall survival curves of patients with lung cancer by MMP1 genotypes**. The individual carriers of at least one 2G allele for MMP1 do not show better survival than the individual with 1G/1G homozygous genotype.

The Kaplan-Meier survival analysis in individual carriers of the 2G allele for MMP1 not show a poorer survival statistically significant (Figure 1). In addition, the multivariate analysis was used to delineate significant prognostic factors for survival. This analysis showed that the MMP1 2G allele was not an independent prognostic factor for overall survival after adjustment for clinical stages and metastases (adjusted HR = 1.23; 95% CI = 0.93–1.63; *P *= 0.153) (Table 4) and individuals with the 2G allele does not show a higher relative risk of death than the individuals with the 1G/1G genotype.

**Table 4:** Analysis of polymorphisms and lung cancer overall survival

**MMPs**	**Genotype**	Adjusted* HR **[95% CI]**	**P**
MMP1	1G/1G	Reference	
	1G/2G	1.25 [0.93–1.69]	0.143
	2G/2G	1.19 [0.85–1.66]	0.312
	1G + 2G	1.23 [0.93–1.63]	0.153
			
MMP8	C/C	Reference	
	C/G + G/G	0.94 [0.68–1.28]	0.675
			
MMP13	A/A	Reference	
	A/G	0.91 [0.68–1.22]	0.543
	G/G	1.53 [0.95–2.47]	0.078
	A/G + G/G	0.97 [0.76–1.23]	0.803

*Adjusted by clinical stages and metastases

**Figure 2:**
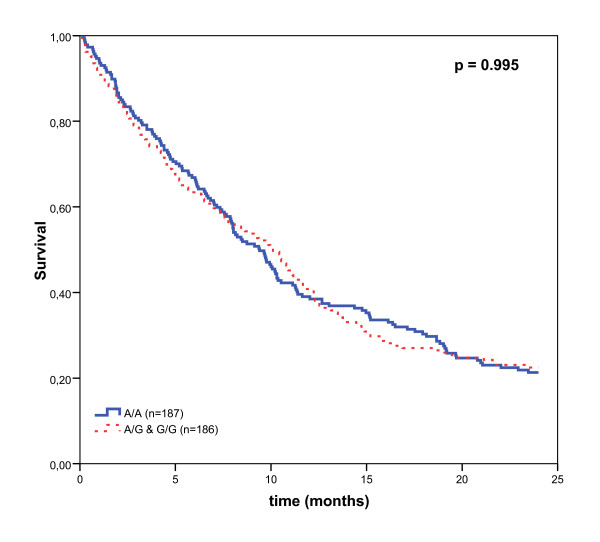
**Kaplan-Meier overall survival curves of patients with lung cancer by MMP13 genotypes**. The individual carriers of at least one G allele for MMP13 do not show better survival than the individual with A/A homozygous genotype.

On the other hand, the analysis of the association between the A/G polymorphism in the MMP13 promoter and survival time revealed that individual carriers of the G allele for MMP13 showed no significant differences in survival time (Figure 2) and the multivariate analyses using Cox's proportional hazard regression analysis did not show statistically significant results (adjusted HR = 0.97; 95% CI = 0.76–1.23) (Table 4).

**Figure 3:**
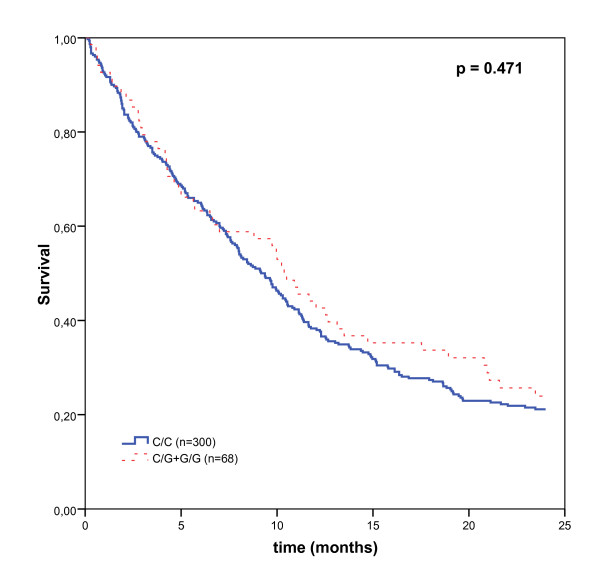
**Kaplan-Meier overall survival curves of patients with lung cancer by MMP8 genotypes**. The individual carriers of at least one G allele for MMP8 do not show better survival than the individual with C/C homozygous genotype.

Finally, although the G allele in MMP8 showed a protective effect against the development of lung cancer, it did not show a statistically significant protective effect associated with the survival time (*P *= 0.675). However, patients who are carriers of at least one G allele not show a better overall survival than the individuals with the C/C homozygous genotype, although a larger sample size would be necessary to confirm these differences (Figure 3). The multivariate analyses using Cox's proportional hazard regression analysis did not show statistically significant results (adjusted HR = 0.94; 95% CI = 0.68–1.28) (Table 4).

## Discussion

In this study we have investigated the effect of polymorphisms in the promoter regions of the three human collagenases (MMP1, MMP8 and MMP13) and the individual risk of developing lung cancer in a group of 501 cases and 510 controls. We have evaluated whether these polymorphisms could influence the progression and survival of these lung cancer patients. Our results suggest that the studied polymorphism in the promoter region of the MMP8 gene is associated with lung cancer risk. Thus, individuals with at least one G variant allele showed a protective effect against developing lung cancer compared to the reference genotype, while this polymorphism not seems associated with a higher survival rate and a better prognosis. On the other hand, the studied polymorphisms in the MMP1 and MMP13 genes do not seem to influence the individual risk to develop lung cancer and survival time in our population.

Our study has several strengths, including high participation of eligible cases (rate 93.8%) and quite large sample size from a homogeneous population of similar ancestry (501 cases and 510 controls). Likewise, all our cases were pathologically confirmed and finally we applied a severe quality control for genotyping. The main limitations of our study were hospital-based subjects, recall bias due to the fact that information on smoking exposure was obtained retrospectively, and especially possible false positive associations, due to the multiple comparisons made. We cannot exclude the possibility that some of these associations may represent chance finding, because the power to detect interactions was limited. To minimize selection bias, we carefully selected controls from patients admitted for various diagnoses that were thought to be unrelated to exposures of interest. Nevertheless, a recent paper from Campbell *et al*. [36] reported that European populations may display various levels of genetic substructure which may lead to false positive associations due to population stratification. In our study, we controlled for this possibility by matching individuals on the basis of European ancestry.

Several reports have showed that proteases from the MMP family are implicated in tumor invasion and metastasis due to their ability to degrade numerous components of the extracellular matrix and basement membrane [37-39]. Focusing on lung cancer, upregulation of certain MMPs, like MMP1 and MMP13, has been associated with the progression and poorer prognosis of squamous cell carcinoma and adenocarcinoma [40-43], suggesting that changes affecting the expression level of these genes could contribute to the progression of this disease. The polymorphisms analyzed in this study have been previously shown to modify the transcriptional activity of the corresponding MMPs [25,44,45].

The 2G allele of the 1G/2G polymorphism in the MMP1 promoter creates an extra Ets-binding site, which results in increased transcriptional activity of this gene [44]. Thus, the presence of the MMP1 polymorphism has been associated with an increased risk of developing different human cancers including colorectal, renal and head and neck [17,21,46]. For lung cancer, the number of studies is still very limited. Recent studies carried out in Caucasian and Asian populations found an association between the 2G polymorphism in the MMP1 promoter and an increased risk of developing lung cancer [47,48]. However, and in agreement with our results, other reports studying the 1G/2G polymorphism in larger populations have not found an increased risk for developing lung cancer for the 2G allele, although stratified analysis by various variables showed statistically significant associations [28,49,50].

Although polymorphisms in MMP genes have been studied extensively with regard to risk of cancer, much less is known about survival outcomes. Studies in other cancers are provocative and suggest that some MMP polymorphisms may have a prognostic role. Accordingly, the 2G allele of the MMP1 1G/2G polymorphism has been associated with worse survival among patients with colorectal [24] and ovarian cancer [23]. However, a study in patients with stage I for NSCLC carrying the variant 2G allele not showed an association between this polymorphism and survival time [51]. Our results do not suggest an association between this polymorphism and decreased survival and this polymorphism is not an independent prognostic factor of overall survival.

On the other hand, the polymorphism -77 A/G in the promoter region of MMP13, which modifies a PEA3 binding site, results in a reduced transcriptional activity of this gene [25], which might contribute to reducing the risk of developing cancer. However, most of the epidemiological studies do not support this biological evidence. Two recent studies of different types of cancer have shown no association between the -77 A/G polymorphism and the risk of developing breast and nasopharyngeal cancer [26,27]. Our results constitute the first report that analyze this polymorphism in lung cancer and suggest that the -77 A/G polymorphism in MMP13 does not contribute to the susceptibility to develop lung cancer in the Asturian population. Thus, the MMP13 polymorphism does not seem to contribute to initial stages of tumor development, although this evidence needs to be confirmed in further studies. Furthermore, this polymorphism doesn't appear to have an effect on patient survival and the multivariate analyses using Cox's proportional hazard regression analysis did not show statistically significant results. These results don't support the biological evidence that MMP13 is implicated in tumor growth and dissemination. However, these findings need to be verified in larger clinical studies.

In the stratified analysis by histological types, we observed that homozygotes for variant allele of polymorphisms in MMP1 and MMP13 genes increase the risk of developing small cell carcinoma. Previous studies have shown that between 70–100% of small lung carcinomas express MMP13 and between 60–70% express MMP1 [52], which suggests that metalloproteinases could be involved in the initial stages of developing this histological types, although the molecular mechanism by which they could participate in this process is still unknown.

Contrary to results observed for polymorphisms in MMP1 and MMP13, the polymorphism studied in MMP8 was associated with a reduced individual susceptibility to develop lung cancer in our study. MMP8 cleaves type I collagen very efficiently and is predominantly expressed and stored in polymorphonuclear (PMN) leukocytes. The physiological role of MMP8 is, however, still unknown. Recent studies in animal models have shown that mutant mice deficient in MMP8 are more susceptible to develop skin cancer, predicting that MMP8 has a protective function against tumor developments [29,30]. In addition, a direct anti-metastatic role for MMP8 was confirmed by Montel et al., who found that overexpression of MMP8 in breast cancer cell lines decreased metastases, which suggests that a greater expression of MMP8 could result in a lower incidence of cancer and a better prognosis [30]. Our data suggest that individual carriers of the allele G in MMP8 had lower susceptibility to develop lung cancer, possibly due to the ability of collagenase-2 to induce anti-metastatic processes. However, these findings need to be verified in larger epidemiological and clinical studies. In the same way, to evaluate gene-gene and gene-environment interactions between the polymorphisms and lung cancer risk and survival time in our population, a single larger sample with thousands of subjects and tissue-specific biochemical and biological characterizations are required.

## Conclusion

In conclusion, this work represents the first study in which polymorphisms in this important group of MMPs (MMP1, MMP8 and MMP13) are analyzed together to understand their contribution to lung cancer development and the effect on disease progression and survival in the Caucasian population. We attempt to contribute to the study of useful biomarkers for screening high-risk populations for primary prevention and early detection of lung cancer. In the same way, as polymorphisms in the promoter region mainly result in changes in expression, it would be interesting to conduct a study of gene expression to confirm our findings. Finally, the biological function of MMPs apparently is more complex than being only involved in growth or tumor progression [53], so further studies would be required to elucidate the molecular mechanisms implicated in these complex processes.

## Competing interests

The authors declare that they have no competing interests.

## Authors' contributions

PGA carried out molecular genetic studies and drafted the manuscript. MFLC participated in the molecular genetic studies and revised the manuscript. AFS performed the statistical analysis. TP and MGM participated in the patient enrollment. XSP participated in the design of the molecular genetic study and revised the manuscript. AT conceived of the study, participated in its design and coordination, and revised the manuscript. All authors read and approved the final manuscript.

## Pre-publication history

The pre-publication history for this paper can be accessed here:



## Supplementary Material

Additional file 1 Multivariate analysis of collagenase-1 (MMP1) stratified by selected variables. This table shows the stratified analysis by selected variables of MMP1 -1607 1G/2G polymorphism. Click here for file

Additional file 2 Multivariate analysis of collagenases and lung cancer risk by histological types. This table shows the stratified analysis by histological types of MMP1, 13 and 8. Click here for file

Additional file 3 Multivariate analysis of collagenase-3 (MMP13) stratified by selected variables. This table shows the stratified analysis by selected variables of MMP13 -77 A/G polymorphism. Click here for file

Additional file 4 Multivariate analysis of collagenase-2 (MMP8) stratified by selected variables. This table shows the stratified analysis by selected variables of MMP8 +17 C/G polymorphism. Click here for file
