# Supplementary material for: Polymorphism +17 C/G in Matrix Metalloprotease MMP8 decreases lung cancer risk
Source: BMC Cancer. 2008 Dec 19;8:378. doi: 10.1186/1471-2407-8-378 (PMC2628929; doi:10.1186/1471-2407-8-378)
Supplement: Additional file 1 — Multivariate analysis of collagenase-1 (MMP1) stratified by selected variables. This table shows the stratified analysis by selected variables of MMP1 -1607 1G/2G polymorphism. [file 1471-2407-8-378-S1.doc]

**Additional file 1 - Multivariate analysis of collagenase-1 (MMP1) stratified by selected variables**

| **Variables** | **Cases/ Controls** | | | **Adjusted OR [95% CI]** | | | **P trend** |
| --- | --- | --- | --- | --- | --- | --- | --- |
|  | **1G/1G** | **1G/2G** | **2G/2G** | **1G/1G** | **1G/2G** | **2G/2G** |  |
| Gender^1^ |  |  |  |  |  |  |  |
| Male | 114/103 | 220/ 227 | 107/110 | 1.00 | 0.93 [0.63-1.37] | 0.98 [0.62-1.55] | 0.936 |
| Female | 14/16 | 28/32 | 18/22 | 1.00 | 1.23 [0.45-3.36] | 1.38 [0.47-4.08] | 0.563 |
| Age (years)^2^ |  |  |  |  |  |  |  |
| < 55 | 21/32 | 52/52 | 29/39 | 1.00 | 1.51 [0.66-3.48] | 1.25 [0.52-3.01] | 0.689 |
| 55 – 69 | 49/39 | 101/102 | 55/55 | 1.00 | 0.79 [0.44-1.44] | 0.88 [0.45-1.71] | 0.747 |
| ≥ 70 | 58/48 | 95/101 | 41/38 | 1.00 | 0.86 [0.49-1.51] | 1.17 [0.56-2.44] | 0.778 |
| Smoking status^3^ |  |  |  |  |  |  |  |
| Never | 10/31 | 16/67 | 9/38 | 1.00 | 0.72 [0.25-2.10] | 0.81 [0.25-2.64] | 0.747 |
| Ever | 118/ 88 | 232/192 | 116/94 | 1.00 | 0.84 [0.59-1.18] | 0.93 [0.62-1.41] | 0.741 |
| Former | 53/47 | 109/118 | 49/50 | 1.00 | 0.79[0.49-1.29] | 0.97 [0.54-1.74] | 0.891 |
| Current ^*^ | 62/40 | 121/64 | 66/40 | 1.00 | 1.08 [0.63-1.83] | 1.05 [0.57-1.90] | 0.887 |
| Family history of cancer^4^ |  |  |  |  |  |  |  |
| No | 79/77 | 120/151 | 63/76 | 1.00 | 0.83 [0.53-1.30] | 0.95 [0.56-1.60] | 0.807 |
| Lung cancer | 10/4 | 35/20 | 12/11 | 1.00 | 0.47 [0.07-2.99] | 0.15 [0.02-1.11] | 0.039 |
| Other cancer | 30/33 | 73/78 | 41/36 | 1.00 | 1.47 [0.74-2.90] | 1.81 [0.84-3.93] | 0.137 |

^1^ Odds ratios (ORs) adjusted by age, family history of cancer, and tobacco consumption (in pack-years)

^2^ Odds ratios (ORs) adjusted by gender, family history of cancer, and tobacco consumption (in pack-years)

^3^ Odds ratios (ORs) adjusted by gender, age, and family history of cancer

^4^ Odds ratios (ORs) adjusted by gender, age, and tobacco consumption (in pack-years)

^5^ Odds ratios (ORs) adjusted by gender, age, family history of cancer, and tobacco consumption (in pack-years)

^*^ Former ≥ 1 year are included
