# Supplementary material for: Polymorphism +17 C/G in Matrix Metalloprotease MMP8 decreases lung cancer risk
Source: BMC Cancer. 2008 Dec 19;8:378. doi: 10.1186/1471-2407-8-378 (PMC2628929; doi:10.1186/1471-2407-8-378)
Supplement: Additional file 2 — Multivariate analysis of collagenases and lung cancer risk by histological types. This table shows the stratified analysis by histological types of MMP1, 13 and 8. [file 1471-2407-8-378-S2.doc]

**Additional file 2 - Multivariate analysis of collagenases and lung cancer risk by histological types**

| **Gene** | **Squamous cell carcinoma** | | **Adenocarcinoma** | | **Small cell carcinoma** | |
| --- | --- | --- | --- | --- | --- | --- |
|  | **Cases/Controls** | **OR [95% CI]^1^** | **Cases/ Controls** | **OR [95% CI]^1^** | **Cases/ Controls** | **OR [95% CI]^1^** |
| MMP1 |  |  |  |  |  |  |
| 1G/1G | 48/119 | 1.00 | 42/119 | 1.00 | 14/119 | 1.00 |
| 1G/2G | 106/259 | 1.08 [0.67-1.76] | 64/259 | 0.79 [0.48-1.30] | 38/259 | 1.44 [0.69-2.99] |
| 2G/2G | 48/132 | 1.09 [0.62-1.93] | 37/132 | 0.85 [0.48-1.51] | 29/132 | **2.06 [0.94-4.51]** |
| MMP13 |  |  |  |  |  |  |
| A/A | 108/267 | 1.00 | 72/267 | 1.00 | 31/267 | 1.00 |
| A/G | 76/201 | 1.09 [0.72-1.66] | 61/201 | 1.23 [0.80-1.91] | 40/201 | **1.94 [1.09-3.45]** |
| G/G | 18/42 | 1.03 [0.51-2.10] | 10/42 | 0.91 [0.40-2.05] | 10/42 | **2.38 [1.01-5.65]** |
| MMP8 |  |  |  |  |  |  |
| C/C | 161/358 | 1.00 | 110/358 | 1.00 | 67/358 | 1.00 |
| C/G+G/G | 39/118 | **0.51 [0.31-0.84]** | 29/118 | 0.79 [0.48-1.31] | 12/118 | **0.43 [0.20-0.89]** |

^1^Odds ratios (ORs) adjusted by gender, age, family history of cancer, and tobacco consumption (in pack-years)
