# Supplementary material for: Polymorphism +17 C/G in Matrix Metalloprotease MMP8 decreases lung cancer risk
Source: BMC Cancer. 2008 Dec 19;8:378. doi: 10.1186/1471-2407-8-378 (PMC2628929; doi:10.1186/1471-2407-8-378)
Supplement: Additional file 3 — Multivariate analysis of collagenase-3 (MMP13) stratified by selected variables. This table shows the stratified analysis by selected variables of MMP13 -77 A/G polymorphism. [file 1471-2407-8-378-S3.doc]

**Additional file 3 - Multivariate analysis of collagenase-3 (MMP13) stratified by selected variables**

| **Variables** | **Cases/ Controls** | | | **Adjusted OR [95% CI]** | | | **P trend** |
| --- | --- | --- | --- | --- | --- | --- | --- |
|  | **A/A** | **A/G** | **G/G** | **A/A** | **A/G** | **G/G** |  |
| Gender^1^ |  |  |  |  |  |  |  |
| Male | 214/226 | 185/175 | 42/39 | 1.00 | 1.21 [0.86-1.70] | 1.23 [0.70-2.16] | 0.272 |
| Female | 34/41 | 23/26 | 3/3 | 1.00 | 1.22 [0.53-2.82] | 0.80 [0.10-6.46] | 0.813 |
| Age (years)^2^ |  |  |  |  |  |  |  |
| < 55 | 50/61 | 43/52 | 9/10 | 1.00 | 1.32 [0.66-2.66] | 1.44 [0.43-4.79] | 0.400 |
| 55 – 69 | 109/99 | 81/79 | 15/18 | 1.00 | 0.99 [0.61-1.60] | 0.91 [0.39-2.12] | 0.854 |
| ≥ 70 | 89/107 | 84/70 | 21/14 | 1.00 | 1.63 [0.97-2.76] | 1.65 [0.68-4.01] | 0.074 |
| Smoking status^3^ |  |  |  |  |  |  |  |
| Never | 20/68 | 13/59 | 2/9 | 1.00 | 0.65 [0.26-1.58] | 0.80 [0.13-5.11] | 0.433 |
| Ever | 228/199 | 195/142 | 43/33 | 1.00 | 1.24 [0.92-1.68] | 1.26 [0.75-2.13] | 0.163 |
| Former | 100/124 | 86/71 | 25/20 | 1.00 | **1.60 [1.05-2.45]** | 1.76 [0.89-3.47] | 0.022 |
| Current ^*^ | 125/68 | 106/64 | 18/12 | 1.00 | 0.96 [0.61-1.52] | 0.85 [0.36-2.00] | 0.729 |
| Family history of cancer^4^ |  |  |  |  |  |  |  |
| No | 122/155 | 115/124 | 25/25 | 1.00 | 1.29 [0.87-1.92] | 1.22 [0.63-2.37] | 0.276 |
| Lung cancer | 34/15 | 20/17 | 3/3 | 1.00 | 0.48 [0.14-1.59] | 0.89 [0.07-10.70] | 0.393 |
| Other cancer | 73/84 | 58/53 | 13/10 | 1.00 | 1.44 [0.82-2.55] | 1.49 [0.53-4.20] | 0.213 |

^1^ Odds ratios (ORs) adjusted by age, family history of cancer, and tobacco consumption (in pack-years)

^2^ Odds ratios (ORs) adjusted by gender, family history of cancer, and tobacco consumption (in pack-years)

^3^ Odds ratios (ORs) adjusted by gender, age, and family history of cancer

^4^ Odds ratios (ORs) adjusted by gender, age, and tobacco consumption (in pack-years)

* Former ≥ 1 year are included
