# Supplementary material for: Polymorphism +17 C/G in Matrix Metalloprotease MMP8 decreases lung cancer risk
Source: BMC Cancer. 2008 Dec 19;8:378. doi: 10.1186/1471-2407-8-378 (PMC2628929; doi:10.1186/1471-2407-8-378)
Supplement: Additional file 4 — Multivariate analysis of collagenase-2 (MMP8) stratified by selected variables. This table shows the stratified analysis by selected variables of MMP8 +17 C/G polymorphism. [file 1471-2407-8-378-S4.doc]

**Additional file 4 - Multivariate analysis of collagenase-2 (MMP8) stratified by selected variables**

| **Variables** | **Cases/ Controls** | | **Adjusted OR [95% CI]** | | **P trend** |
| --- | --- | --- | --- | --- | --- |
|  | **C/C** | **C/G + G/G** | **C/C** | **C/G + G/G** |  |
| Gender^1^ |  |  |  |  |  |
| Male | 344/311 | 88/100 | 1.00 | **0.63 [0.43-0.93]** | **0.021** |
| Female | 48/47 | 12/18 | 1.00 | 0.71 [0.27-1.87] | 0.482 |
| Age (years)^2^ |  |  |  |  |  |
| < 55 | 76/88 | 22/31 | 1.00 | 0.62 [0.28-1.34] | 0.220 |
| 55 – 69 | 164/141 | 37/40 | 1.00 | 0.72 [0.40-1.29] | 0.270 |
| ≥ 70 | 152/129 | 41/47 | 1.00 | 0.65 [0.36-1.16] | 0.144 |
| Smoking status^3^ |  |  |  |  |  |
| Never | 28/94 | 7/28 | 1.00 | 0.69 [0.24-1.98] | 0.487 |
| Ever | 364/264 | 93/90 | 1.00 | **0.69 [0.48-0.97]** | **0.034** |
| Former | 166/145 | 40/55 | 1.00 | **0.63 [0.38-1.02]** | **0.059** |
| Current^*^ | 192/109 | 53/31 | 1.00 | 0.83 [0.49-1.43] | 0.511 |
| Family history of cancer^4^ |  |  |  |  |  |
| No | 205/217 | 52/68 | 1.00 | 0.77 [0.48-1.22] | 0.267 |
| Lung cancer | 49/22 | 8/8 | 1.00 | **0.14 [0.03-0.64]** | **0.011** |
| Other cancer | 112/103 | 29/36 | 1.00 | 0.60 [0.32-1.14] | 0.119 |

^1^ Odds ratios (ORs) adjusted by age, family history of cancer, and tobacco consumption (in pack-years)

^2^ Odds ratios (ORs) adjusted by gender, family history of cancer, and tobacco consumption (in pack-years)

^3^ Odds ratios (ORs) adjusted by gender, age, and family history of cancer

^4^ Odds ratios (ORs) adjusted by gender, age, and tobacco consumption (in pack-years)

*Former ≥ 1 year are included
